# Efficient Alcoholysis of Poly(ethylene terephthalate) by Using Supercritical Carbon Dioxide as a Green Solvent

**DOI:** 10.3390/polym16111564

**Published:** 2024-05-31

**Authors:** Yiwei Xu, Ran Cui, Yuqing Han, Jie Jiang, Dongdong Hu, Ling Zhao, Zhenhao Xi

**Affiliations:** 1 State Key Laboratory of Chemical Engineering, School of Chemical Engineering, East China University of Science and Technology, Shanghai 200237, China; xyw990610@126.com (Y.X.); cuiran@mail.ecust.edu.cn (R.C.); hanyuqing0729@163.com (Y.H.); jiangjie@ecust.edu.cn (J.J.); zhaoling@ecust.edu.cn (L.Z.); 2 Shanghai Key Laboratory of Multiphase Materials Chemical Engineering, East China University of Science and Technology, Shanghai 200237, China; hudd@ecust.edu.cn

**Keywords:** poly(ethylene terephthalate), alcoholysis, supercritical carbon dioxide, molecular dynamics simulation

## Abstract

In order to reduce the environmental impact of poly(ethylene terephthalate) (PET) plastic waste, supercritical fluids were used to facilitate effective recovery via improved solvent effects. This work focuses on the mechanisms of supercritical CO_2_ (ScCO_2_) during the alcoholysis processing of PET using systematic experiments and molecular dynamics (MD) simulations. The results of the alcoholysis experiment indicated that PET chips can be completely depolymerized within only an hour at 473 K assisted with ScCO_2_ at an optimal molar ratio of CO_2_/ethanol of 0.2. Random scission of PET dominates the early stage of the depolymerization reaction process, while specific scission dominates the following stage. Correspondingly, molecular dynamics (MD) simulations revealed that the solubilization and self-diffusion properties of ScCO_2_ facilitate the transportation of alcohol molecules into the bulk phase of PET, which leads to an accelerated diffusion of both oligomers and small molecules in the system. However, the presence of excessive CO_2_ has a negative impact on depolymerization by weakening the hydrogen bonding between polyester chain segments and ethanol, as well as decreasing the swelling degree of PET. These data provide a deep understanding of PET degradation by alcohols and the enhancement of ScCO_2_. It should be expected to achieve an efficient and high-yield depolymerization process of wasted polyesters assisted with ScCO_2_ at a relatively low temperature.

## 1. Introduction

PET is widely used in a range of applications, including food packaging, textiles, films, and sheets, due to its adaptability, durability, and low cost [1,2]. Worldwide PET production grew from 42 million tons in 2014 to 87 million tons in 2022 [3]. Nevertheless, the excellent chemical stability of PET poses considerable obstacles in terms of spontaneous decomposition. Large-scale PET production and its non-degradability result in massive accumulation in nature. Meanwhile, most waste PET is disposed of via incineration or landfill, causing a wide range of environmental and sustainability issues. Therefore, effective and economical recycling measures are essential [4].

The chemical depolymerization of PET into monomers has been found to be possible and cost-effective [5,6]. PET chemical recycling processes include alcoholysis, glycolysis, aminolysis, hydrolysis, and various other methods [7]. Among them, the products produced through alcoholysis and glycolysis are notable for their elevated purity, milder reaction conditions, and the ability to be manufactured on an industrial scale [8]. However, conventional chemical recycling methods are inefficient under solid state conditions at relatively low temperatures but susceptible to generating by-products in a molten state at high temperatures [9]. Performing the complete depolymerization of PET with a high yield, such as via glycolysis, is a difficult task to accomplish, even when using catalysts.

In recent years, there has been significant interest in depolymerization assisted with supercritical fluids due to their outstanding physicochemical properties, particularly their excellent dissolving and diffusion capacities [10,11]. When supercritical methanol was utilized during the depolymerization of molten PET at a temperature of 573 K and over 10 MPa, the yield of dimethyl terephthalate (DMT) was up to 80% in about 30 min [12]. In addition, under supercritical conditions, ethanol [13,14,15], as well as ethylene glycol [16], propylene glycol [17], and iso-octanol [18,19], can also accelerate the depolymerization process of PET in a molten state. Nevertheless, the supercritical alcoholysis process of molten PET is usually harsh and energy-intensive, with temperatures typically exceeding 573 K and pressures over 10 MPa. The challenge is to develop an efficient depolymerization process for wasted polyesters with milder supercritical conditions while reducing possible side reactions.

However, when carbon dioxide and alcohol are combined, the fluid mixture has a relatively lower critical point while simultaneously improving diffusion [20,21], which may enhance the depolymerization process under milder conditions. Liu et al. [22] obtained 95% DMT yield in only 40 min via the methanol depolymerization of PET by using supercritical CO_2_ at 543 K and 10 MPa. Yu et al. [23] found the optimal depolymerization conditions for PET/PE at 543 K, with both ethanol and CO_2_ at an initial injection pressure of 2.0 MPa. Li and Guo et al. [24,25] conducted PET hydrolysis using a solid acid catalyst in ScCO_2_ and proposed a depolymerization mechanism wherein water molecules and hydrated hydrogen ions enter the non-crystalline area of PET through the dissolution of ScCO_2_ until achieving the complete hydrolysis of PET. In conjunction with experiments, Spyriouni et al. [26] modeled the adsorption isotherms of supercritical CO_2_ in polystyrene and discovered that increasing temperature and pressure enhanced polystyrene swelling. Bozorgmehr et al. [27] used MD simulations to study the interaction of polyethylene with water, ethanol, and methanol in supercritical conditions at the molecular level, and the results were compatible with the experimental depolymerization of polyethylene in a relative binary system. Sun et al. [28] used molecular dynamics simulations to investigate PET’s glass transition behavior and fractional free volume (FFV) in a ScCO_2_ environment. Nevertheless, few studies have integrated macroscopic and microscopic viewpoints to explain how supercritical fluids enhance depolymerization in ternary systems.

Therefore, this work aims to investigate the alcoholysis process of PET in a solid state assisted with supercritical CO_2_. The depolymerization behavior, as well as the corresponding mechanism, will be studied by combining molecular dynamics simulations and experiments. The changes in molecular weight distribution, as well as the depolymerization conversion of PET and the yield of diethyl terephthalate (DET), were quantitatively analyzed with changes in CO_2_ content, alcohol type, temperature, and reaction time. Correspondingly, the depolymerization mechanism and enhancement principle were comparatively demonstrated through microscopic computations on the radial distribution functions, self-diffusion coefficients, solvent-accessible surface areas, and hydrogen bonding interactions in both binary alcoholysis and ternary systems with supercritical CO_2_, respectively.

## 2. Materials and Methods

### 2.1. Materials

Raw PET material was supplied by Lotte Chemical (Shanghai, China). PET possesses a density of 1.4 g/cm^3^, an average molecular weight of 12,600 g/mol, and a melting point of 533 K. PET pellets were smashed through a 10–50 mesh and subsequently dehydrated under a vacuum before being utilized. Adamas (Shanghai, China) supplied methanol (AR, 99.5%), ethanol (GR, 99.8%), n-propanol (AR, 99.5%), n-butanol (AR, 99.5%), and n-pentanol (GC, 99%). CO_2_ (a purity of 99.99%) was purchased from Air Liquide (Shanghai, China). The catalyst, anhydrous zinc acetate (AR, 99%), was provided by Macklin (Shanghai, China). DET (AR, 99%) was supplied by Tischiai Chemical Industry Development Co. (Shanghai, China).

### 2.2. Experimental Process

The following instruments were utilized in this depolymerization experiment: a temperature sensor, a time sensor, a pressurization system, an oil bath heating system, and a reaction vessel. The reaction kettle has a capacity of 120 mL and is designed to withstand a pressure of 30 MPa. The pressure inside the vessel was accurately monitored using a pressure sensor with a measurement precision of 0.1 MPa. A temperature control system with a precision of 0.5 K regulated the temperature within the reaction vessel. Two temperature sensors attached to the temperature control system detected the temperature inside the reaction kettle and the temperature of the oil bath, respectively. Prior to the reaction, 5 g of PET pellets, 0.05 g of anhydrous zinc acetate, and 0.65 moles of alcohol reagent were introduced into the reaction kettle and sealed. Subsequently, a CO_2_ dispenser (SFT-10, Septech Ltd., Shanghai, China) was employed to introduce a specific volume of CO_2_ at a constant rate (200 mL/h) under the environment’s conditions. The molar ratio of CO_2_ to alcohol was maintained in the range of 0–1.0. After the oil bath was preheated to the designated reaction temperature, the reaction vessel was immersed in it to initiate the reaction and timed accordingly. Once the oil bath was preheated to temperatures of 453, 473, 493, and 513 K, the vessel was immersed in it and left for a duration of 30 min. When evaluating the effect of reaction time, the oil bath was heated to a temperature of 473 K with varying reaction times: 20, 30, 40, 50, and 60 min. All of the experimental groups were conducted in parallel and independently. After the depolymerization reaction was completed, the container holding the reaction mixture was cooled down using an ice–water bath. Correspondingly, the pressure inside the container was then gradually reduced to atmospheric pressure by slowly draining the contents within 10 min. Ultimately, the resulting compounds were separated in both liquid and solid states after filtration and collection. The solid states were primarily unreacted PET, while the phase states were predominantly DET [29].

### 2.3. Characterization

The residual solid-phase product after the depolymerization process was washed repeatedly with ethanol before being placed in a vacuum drying oven and dried under vacuum at 373 K for 24 h to a constant weight. The conversion of PET was calculated as follows,

(1) $$\mathrm{Conversion}\;\mathrm{of}\;\mathrm{PET}=\frac{w_{0}-w_{1}}{w_{0}}\times 100\%$$

where *w*_0_ and *w*_1_ are the mass of the initial PET and the residual product, respectively. Correspondingly, the molecular weight distributions of the residual solid-phase products were determined using a Varian PL-GPC 50 instrument. A PL HFIPgel column and a RI refractive index detector were used in the system. The column was set to a temperature of 313 K. The mobile phase was Hexafluoroisopropanol (HFIP) and 20 mM sodium trifluoroacetate (NaTFAc) at a flow rate of 1 mL/min.

Ultra-high-performance liquid tandem ultra-high resolution mass spectrometry (UPLC-MS) on an Agilent A1100 equipped with a C18 column and a Trap XCT mass spectrometer was used to identify the components of the liquid-phase products. The major product, DET, was quantitatively assessed using an external standard method on a reversed-phase high-performance liquid chromatograph (RP-HPLC). The RP-HPLC was outfitted with a C18 column and a UV detector set at 254 nm, and the mobile phase was an 80/20 (*V*/*V*) acetonitrile/water solution at a flow rate of 1 mL/min. The column temperature was set at room temperature, and the final yield of the primary product, DET, was computed as follows:

(2) $$\mathrm{Yield}\;\mathrm{of}\;\mathrm{DET}=\frac{\mathrm{Mass}\;\mathrm{of}\;\mathrm{DET}\;\mathrm{production}\;\mathrm{after}\;\mathrm{reaction}}{\mathrm{Theoretical}\;\mathrm{content}\;\mathrm{of}\;\mathrm{DET}\;\mathrm{in}\;\mathrm{waste}\;\mathrm{PET}}\times 100\%$$

### 2.4. Simulation Details

Materials Studio was used to create the molecule structures for PET, CO_2_, and different alcohols, respectively. All of the simulation operations and data analysis were performed by Gromacs 2020 [30]. The CO_2_ molecules were modeled using the TraPPE [31] force field, while the alcohols were modeled using the TraPPE-UA [32] model. Sobtop and Multiwfn were used to model the force field characteristics of PET long-chain polymers under the GAFF force field and applied RESP atomic charges [33,34,35]. The Lorentz–Berthelot rule was used to define the interatomic LJ parameters [36,37].

The temperature was maintained using a velocity-rescaling thermostat [38] with a time constant of *τ_T_* = 0.2 ps. Pressure control for the equilibrium phase was maintained using a Berendsen barostat [39] with a time constant of *τ_P_* = 0.5 ps, while pressure control for the production phase was maintained using a Parrinello–Rahman barostat [40] with a time constant of *τ_P_* = 2.5 ps. Equations of motion were integrated using the leapfrog method, and energy minimization was accomplished using the conjugate gradient method. The cutoff radius for all inter-particle interactions was set to 1.4 nm, the PME algorithm [41] was used for long-range electrostatic interactions, the LINCS algorithm [42] was used to constrain bond lengths and isolated bond angles, and VMD 1.9.3 was used for molecular graph visualization and part of the trajectory processing analysis [43]. Packmol [44] was used to place six polymer chains with 100 repeating units, 600 alcohol molecules, and either 300 or 0 CO_2_ molecules in a 7 nm × 7 nm × 7 nm box. Table 1 displays the composition of the system. The systems were subsequently simulated for 40 ns at 473 K and 100 bar under the NPT ensemble until a stable state was reached. The outcomes were evaluated by subjecting the systems to an additional 20 ns of simulation under the NVT ensemble.

### 2.5. Self-Diffusion Coefficient

To analyze the diffusion behavior of alcohols, CO_2_, and PET, the subsequent equation was employed to compute the mean squared displacement (*MSD*) in the mixture with respect to time,

(3) $$MSD(t)=\frac{1}{N}\sum_{k=1}^{N}\langle |r_{k}(t)-r_{k}(0){|}^{2}\rangle$$

where N is the number of particles, and *r_k_*(*t*) and *r_k_*(0) are the positions of the *k*th particle at the moment *t* and the initial moment, respectively. The self-diffusion coefficient *D* can thus be obtained from Einstein’s formula,

(4) $$D=\frac{1}{6N}\underset{t\to \infty }{lim}\frac{dMSD}{dt}$$

For PET, distinct calculations were performed on the *D* of the end groups and main chains, respectively. The end groups correspond to the atoms at the ends of the chain, while the other groups are regarded as the main chains.

### 2.6. Hydrogen Bonds

CO_2_ and PET were selected as acceptors, while alcohol molecules were regarded as donors. By using the analytical tool included with Gromacs, the average number and lifetime of hydrogen bonds were computed. The hydrogen bond formation circumstances were tuned to a hydrogen donor–acceptor angle of 30° and a donor–acceptor distance of 0.35 nm [45].

## 3. Result and Discussion

### 3.1. Effect of CO_2_-Alcohols Molar Ratio on PET Depolymerization Behavior

**Figure 1:**
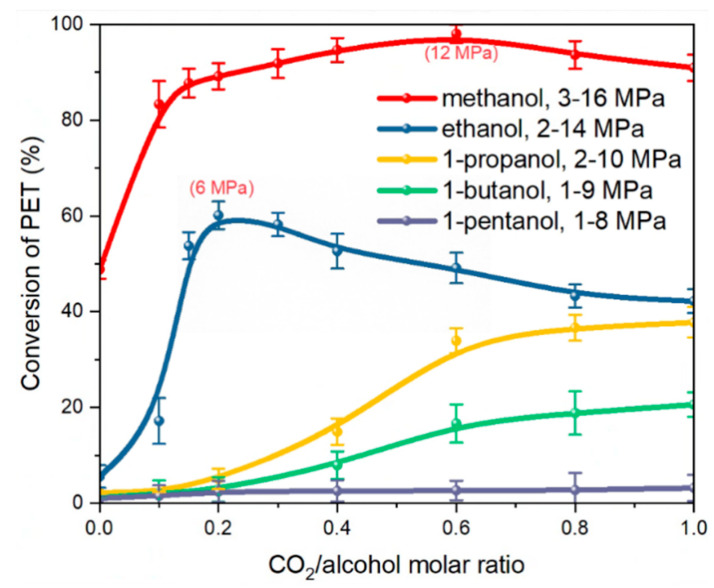
Effect of CO_2_–alcohol molar ratio on the conversion of PET; T = 473 K; t = 30 min.

The depolymerization behavior of PET changed with the use of serious alkyl alcohols with different molar ratios of ScCO_2_ at 473 K, as shown in Figure 1. The results indicated that employing only methanol for PET depolymerization could achieve a reaction conversion rate of approximately 50% with a lack of CO_2_. Meanwhile, the reaction conversion rates obtained with the other alcohols were all below 10%. This is due to the fact that an alcoholysis reaction is a nucleophilic substitution reaction, and the spatial site resistance effect influences the nucleophilic activity of various alcohols [46]. As the volume of the alkyl group on the alcohol molecule increases sequentially, the resulting spatial site-blocking effect also increases, and the activity of the alcohol molecule’s hydroxyl oxygen atom in attacking the carbonyl carbon atom of PET reduces in terms of depolymerization.

After introducing CO_2_ into the system, the reaction conversion of PET depolymerization using various monohydric alcohols improved significantly. This contributed to improving the reactive mass transfer mechanism when using CO_2_. Interestingly, for methanol and ethanol systems, the reaction conversion quickly increased and then gradually decreased with increasing CO_2_ content. The reaction conversion peaked at roughly 98% and 60%, respectively, when the molar ratios of CO_2_ to methanol and ethanol reached 0.6 and 0.2, respectively. This is comparable with the results obtained by Liu [22,23] et al., where CO_2_ was used to promote PET depolymerization with ethanol and methanol. It is predicted that when the partial pressure of carbon dioxide increased, some of the ethanol involved in the reaction dissolved in the carbon dioxide above the phase interface, resulting in a reduction in the amount of ethanol involved in the reaction. However, compared to other monohydric alcohols, the depolymerization of PET with ScCO_2_ and ethanol shows both a faster rate and relatively milder conditions at only 6 MPa. Hence, ethanol was chosen for the subsequent optimization trials.

### 3.2. Effect of the Reaction Time on Depolymerization with CO_2_ and Ethanol

**Figure 2:**
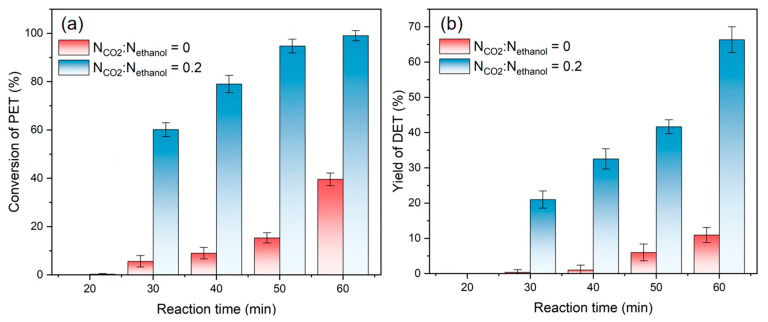
(**a**) Conversion of PET, and (**b**) yields of DET at different reaction times, N_CO2_:N_ethanol_ = 0 and 0.2.

For the PET/CO_2_/ethanol ternary system, depolymerization reaction conversion and DET yield changes with reaction time were tested at molar ratio ranges of CO_2_ to ethanol of 0 and 0.2, with a constant mass ratio of 6 for ethanol to PET at 473 K, as shown in Figure 2. It can be seen that the reaction hardly occurred within the first 30 min without CO_2_. Furthermore, when the reaction time surpassed 60 min, the conversion rate was only approximately 40%. However, the inclusion of CO_2_ resulted in a conversion rate of nearly 100%, with a DET yield of 65%. Observably, the introduction of CO_2_ significantly accelerated the ethanolysis of PET.

**Figure 3:**
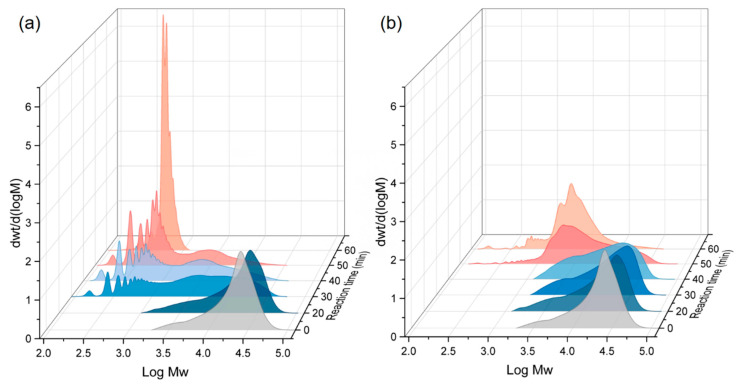
GPC analysis of residual PET at different reaction times, N_CO2_:N_ethanol_ = (**a**) 0.2 and (**b**) 0.

The molecular weight distribution of the residual PET produced was subsequently analyzed for both conditions, and the results are presented in Figure 3 and Table 2. For the PET/ethanol binary system, the average molecular weight of residual PET decreased gradually from 12,586 g/mol to approximately 10,083 g/mol within 30 min and reached approximately 1889 g/mol after 60 min. From 0 to 50 min, the molecular weight of residual PET showed a wide molecular weight dispersion. This indicates that ethanolysis leads to the fragmentation of the polymer chains and, thus, the gradual conversion of large macromolecular chains into smaller chain segments with reduced molecular weights. When CO_2_ was added into the system, the molecular weight of residual PET fell fast to 1143 g/mol after 30 min. CO_2_ expedited the process of ethanol solubilization and diffusion into PET’s bulk phase, leading to the quick decomposition of large molecular chains into smaller chains. Furthermore, the molecular weight distribution of PET progressively broadened during these periods, and multiple peaks were observed in the low-molecular-weight region. This demonstrated that the assault capabilities of ethanol on various ester linkages of PET chains were nearly the same, with the random scission of PET chains dominating. After 60 min, the residual PET was present as a substantial number of oligomers. Monomer yields increased rapidly during this stage, as oligomers decomposed quickly into monomers, and the reactions were driven by specific scission.

### 3.3. Effect of the Reaction Temperature on Depolymerization

**Figure 4:**
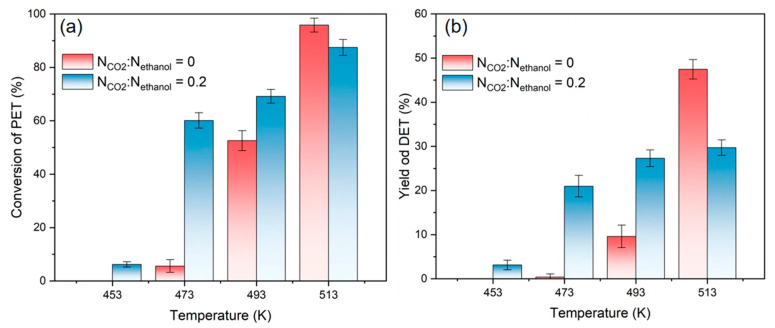
(**a**) Conversion of PET, and (**b**) yields of DET at different reaction temperatures, N_CO2_:N_ethanol_ = 0 and 0.2.

The effect of the reaction temperature on depolymerization at 30 min at molar ratio ranges of CO_2_ to ethanol of 0 and 0.2 and for a weight ratio of ethanol to PET of 6 are summarized in Figure 4. The results showed that the efficiency of PET depolymerization improved gradually with the increasing temperature in the range of 453~493 K. Additionally, the addition of CO_2_ into the system obviously enhances both the reaction conversion and the yield of DET. This is due to the rise in temperature, which enhances the diffusion of molecules in the system, making it easier to dissolve ethanol molecules into PET chain segments when using CO_2_. However, at 513 K, the addition of CO_2_ resulted in poorer reaction conversions and DET yields than those without. In conjunction with the vapor–liquid equilibrium phase diagram of CO_2_ and ethanol [47,48,49], when the temperature is low, CO_2_ is more readily dissolved in ethanol in the liquid phase, but when the temperature is high, ethanol is more readily dissolved in CO_2_. As a result, the amount of ethanol available to react with PET in the liquid phase at high temperatures reduces, making CO_2_ promotion less effective.

### 3.4. Intermolecular Interactions in Ternary Systems of PET-Alcohol-CO_2_

**Figure 5:**
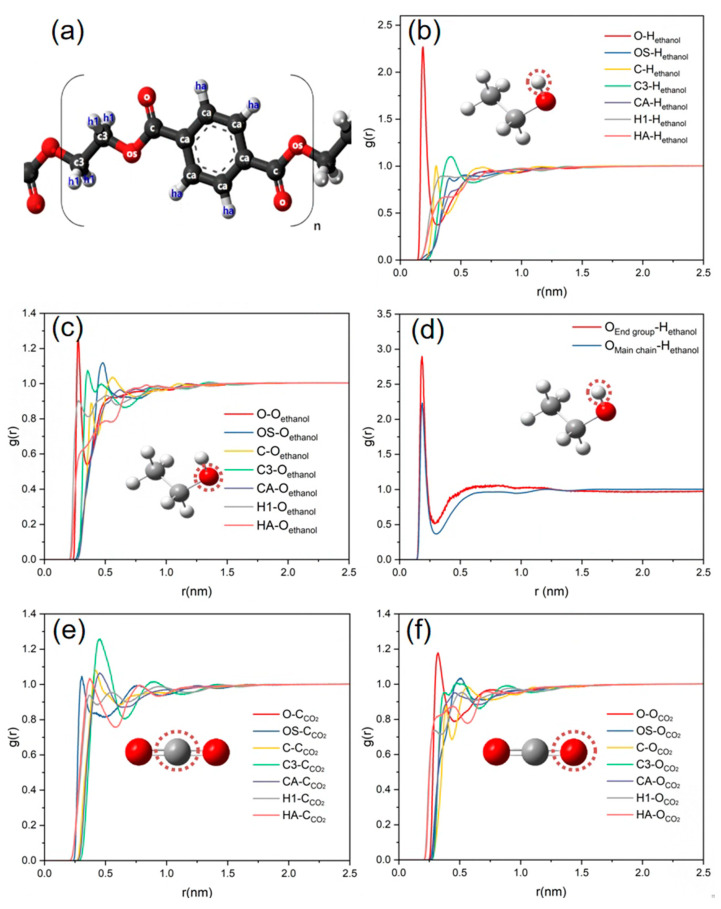
(**a**) The structure of PET. (**b**) RDFs of PET atoms and hydroxyl hydrogen atoms of ethanol. (**c**) RDFs of PET atoms and hydroxyl oxygen atoms of ethanol. (**d**) RDFs of carbonyl oxygen atoms of PET and hydrogen atoms of ethanol. (**e**) RDFs of PET atoms and carbon atom of CO_2_. (**f**) RDFs of PET atoms and oxygen atom of CO_2_.

To explore the interaction behavior between PET and a mixed alcohol–CO_2_ solution, the radial distribution function (RDF) among atoms was calculated using the ternary system CO_2_-PET-ethanol as a model. The first sharp peaks of the O_PET_-H_ethanol_ and O_PET_-O_ethanol_ RDFs occur at about 0.2 nm and 0.25 nm, respectively, which is higher than the RDF peaks between the other atoms on the PET and hydroxyl group of the ethanol molecule (Figure 5b,c). This indicates that the ester groups of PET can form a significant number of hydrogen bonds with ethanol molecules, which means that the carbonyl oxygen atoms of PET can preferentially adsorb the hydroxyl group. The strong hydrogen bonding between the hydroxyl group of ethanol and the carbonyl group of PET is regarded as the active site in the reaction process, enhancing the swelling and dissolution of PET, and eventually accelerating the reaction [50].

RDF analysis was conducted to determine the radial distribution function between the carbonyl oxygen atoms on PET and the ethanol hydroxyl hydrogen atoms at the end group and main chain, which is shown in Figure 5d. Meanwhile, the ones between each atom on the PET chain and the oxygen and carbon atoms on carbon dioxide are shown in Figure 5e,f, respectively. From Figure 5d, it can be seen that both the peaks of O_endgroup_-H_ethanol_ and O_mainchain_-H_ethanol_ RDFs occur at about 0.2 nm, and the peak of O_endgroup_-H_ethanol_ is higher than the other one. Compared to the main chain, the end group of PET should be easily capable of establishing more robust hydrogen bonding interactions with ethanol molecules. In addition, the first peaks of OS_PET_-C_CO2_ and O_PET_-O_CO2_ RDFs occur at about 0.3 nm and 0.32 nm, respectively, as shown in Figure 5e,f. This is due to a weak interaction between the ester group of PET and CO_2_. When double bonds form between carbon and oxygen atoms, CO_2_ often functions as a Lewis base due to the presence of lone pairs of electrons on the oxygen atoms. This has been revealed through the use of FT-IR spectroscopy, with the presence of weakly attractive interactions between CO_2_ and molecules containing carbonyl groups [51]. Based on the Lewis acid effect, the carbonyl oxygen atom on PET enables engaging in LA-LB interactions with CO_2_. This results in a greater dispersion of CO_2_ molecules around the carbonyl group of the PET molecular chain, which enhances the solubility of PET in CO_2_ [52].

In addition, the hydrogen bonding between PET and ethanol molecules, as well as between ethanol and CO_2_ molecules, was calculated in the PET–ethanol–CO_2_ ternary system at various CO_2_ concentrations, as listed in Table 3. The results reveal that as CO_2_ content increases, the average number of hydrogen bonds (HBs) between PET and ethanol molecules decreases, whereas the average number of hydrogen bonds between CO_2_ and ethanol molecules grows until stabilizing. Consequently, the ongoing addition of CO_2_ has the opposite effect of diminishing the hydrogen bonding between ethanol molecules and PET, making it challenging to establish stronger hydrogen connections. This means that CO_2_ and ethanol exhibit a competitive relationship when adsorbed by PET. In reality, ethanol has a greater tendency to dissolve into CO_2_, and excessive CO_2_ should be unfavorable to the depolymerization process, which is consistent with the experimental trend shown in Figure 1.

To compare the differences between various alcohol molecules, the intensity of the hydrogen bonding interaction between PET and different alcohol molecules was also calculated, as shown in Table 4. The results show that the shorter the alkyl chain of the alcohol molecule, the larger the average number of hydrogen bonds generated between the alcohol molecule and the PET chain, and hence, the stronger the hydrogen bonding effect. The introduction of CO_2_ resulted in a general weakening of the hydrogen bonding connections between alcohol molecules and PET.

### 3.5. Diffusion Behaviour

**Figure 6:**
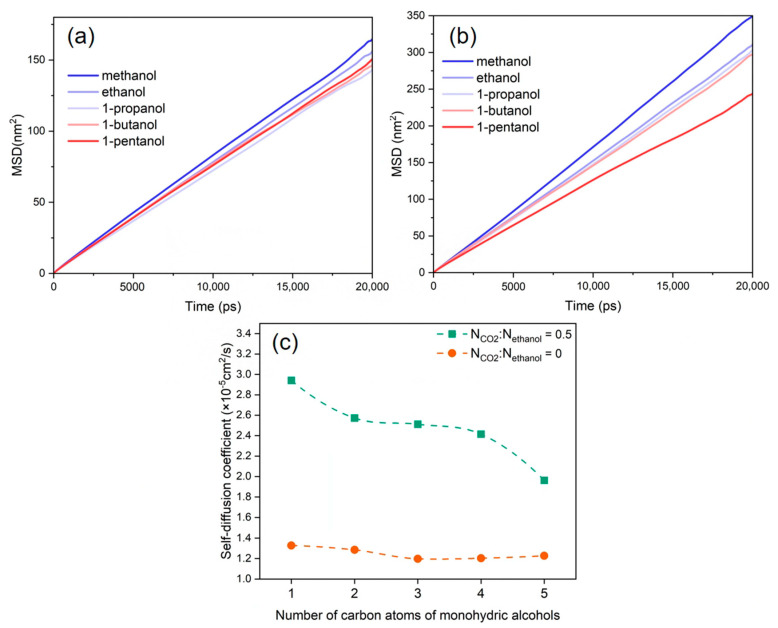
*MSD* of alcohols, N_CO2_:N_alcohol_ is (**a**) 0 and (**b**) 0.5. (**c**) Self-diffusion coefficients of alcohols at different CO_2_ contents.

The diffusion behavior of alcohol molecules plays a crucial role in depolymerization efficiency due to mass transfer limitations during the alcoholysis of PET [53]. The *MSD* distributions and self-diffusion coefficients of the various alcohol molecules with and without the addition of CO_2_ are displayed relatively in Figure 6a–c. The results indicate that the diffusion coefficients of several alcohol molecules in the PET bulk phase system were ranked as follows: methanol > ethanol > 1-propanol > 1-butanol > 1-pentanol. This is also consistent with the depolymerization reaction behavior of PET combined with different alkyl alcohols shown in Figure 1. This is because the molecular volume of alcohol molecules increases with the lengthening of their alkyl chains, limiting their mobility in the PET bulk phase and ultimately lowering the diffusion coefficient.

After the introduction of CO_2_ into the solution, the diffusion coefficients of various alcohol molecules were enhanced, as shown in Figure 6c. The presence of a hydrogen bonding effect between CO_2_ and alcohol molecules enhances the dissolution of alcohol molecules into CO_2_, resulting in the strong diffusion behavior of alcohol molecules [54]. Meanwhile, the length of the alkyl chain influences the enhanced diffusion abilities of various alcohol molecules in distinct ways. The shorter the alkyl chain of alcohol molecules, the smaller the spatial site resistance, the greater the compatibility with CO_2_, and the more noticeable the enhancing effect, which is compatible with the experimental law observed in the CO_2_–alcohol binary system [55].

**Figure 7:**
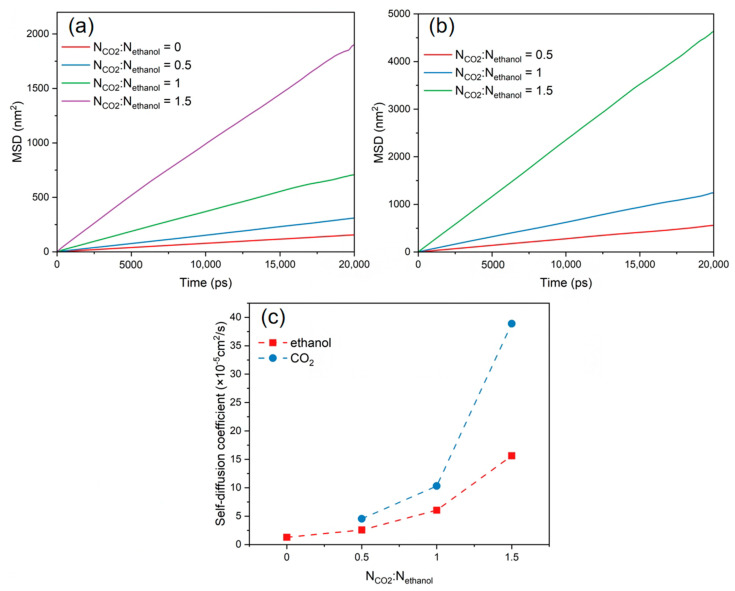
*MSD* of (**a**) ethanol and (**b**) CO_2_ at different CO_2_ contents. (**c**) Self-diffusion coefficients of ethanol and CO_2_ at different CO_2_ contents.

**Figure 8:**
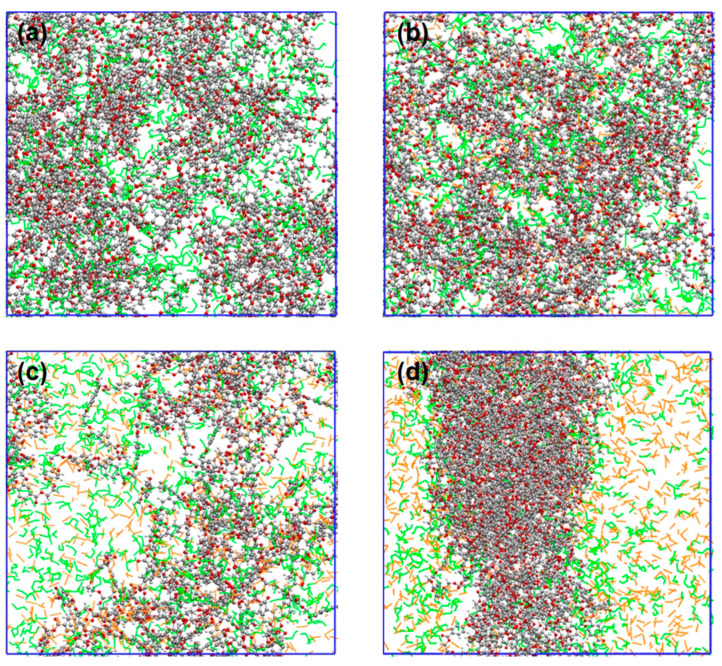
Equilibrated snapshots at different CO_2_ contents, N_CO2_:N_ethanol_ is (**a**) 0, (**b**) 0.5, (**c**) 1, and (**d**) 1.5. Ethanol molecules are labelled in green, CO_2_ molecules are labelled in yellow, and the rest are PET molecules.

The *MSD*s and self-diffusion coefficients of CO_2_ and ethanol molecules were calculated at various CO_2_ concentrations, as shown in Figure 7. These show that the *MSD* profiles and self-diffusion coefficient of CO_2_ and ethanol molecules rose as the CO_2_ content increased. The continual addition of CO_2_ increased hydrogen bonding with alcohol molecules and LA-LB interaction with PET chains, resulting in the faster overall diffusion of the system. When the CO_2_/ethanol molar ratio was 1.5, the self-diffusion coefficient between CO_2_ and ethanol molecules increased significantly. However, in combination with the equilibrium snapshots (Figure 8), it can be found that more ethanol molecules separated from within the PET bulk phase and entered into the CO_2_ solution. This implies that when additional CO_2_ is introduced, ethanol is more likely to dissolve into the CO_2_ rather than being absorbed by the PET chains.

**Figure 9:**
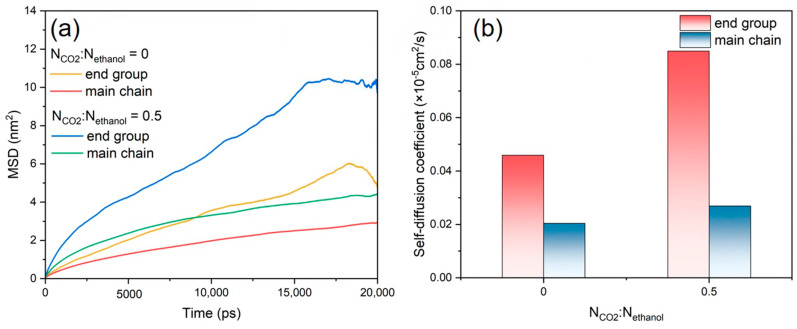
(**a**) *MSD* and (**b**) self-diffusion coefficients of the end groups and main chains of PET, N_CO2_:N_ethanol_ is 0 and 0.5.

In the PET–ethanol–CO_2_ ternary system, further analyses of the variation in the diffusion coefficients of the PET end groups and main chains were conducted, as shown in Figure 9a,b. The *MSD* profiles and diffusion coefficients of the end groups are higher than those of the main chain, as can be seen from the results, suggesting that the end groups move more quickly than the main chain. PET end groups have a quicker diffusion rate than the main chain, which contributes to rapid alcoholysis [56]. Conversely, the introduction of CO_2_ resulted in enhancements in both the *MSD* curves and diffusion coefficients of the PET end groups in relation to the main chain. This is due to the LA-LB interaction between CO_2_ and PET chains, which accelerates the overall movement of PET chains and is one of the primary reasons why CO_2_ improves the PET alcoholysis process.

### 3.6. Swelling Behaviors

**Figure 10:**
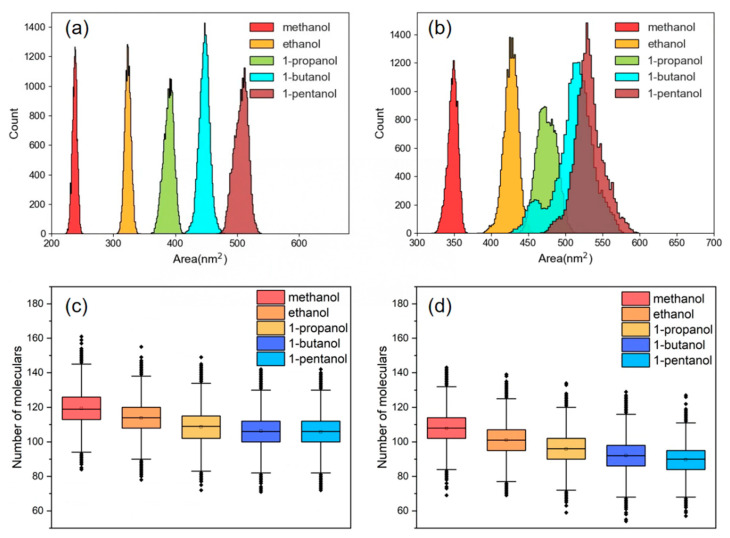
SASA distribution of PET in the system, N_CO2_:N_alcohol_ is (**a**) 0 and (**b**) 0.5. Number distribution of different alcohol molecules at 0.2 nm near PET, N_CO2_:N_alcohol_ is (**c**) 0 and (**d**) 0.5.

When PET chips are placed in a heated solution, the macromolecule cluster gradually expands, and the mobility of the PET chains is enhanced with the increasing free volume between chains. Eventually, the entire PET chain begins to move together and a large number of small molecules gradually enter into the bulk phase voids [57]. The solvent-accessible surface area (SASA) can serve as a measure of polymer conformational changes in a solvent [58]. This is helpful for the calculation of PET chain swelling caused by various alcohol molecules before and after the introduction of CO_2_, as depicted in Figure 10a,b. These findings indicated that the distribution of SASA grows as the length of the alkyl chain of alcohol increases, reflecting the improved swelling effect of the polymer by the alcohol molecules. This aligns with Hirogaki’s principle regarding an expansion ratio of PET, which was assessed by introducing several alcohols as modifiers in a ScCO_2_ atmosphere [59]. After the introduction of CO_2_ into the system, a general increase in the SASA distribution of various alcohol molecules occurred. Alcohol molecules carried by CO_2_ can penetrate PET more easily by expanding the free volume of the PET bulk phase and increasing the activity of molecular chain segments.

Since the carbonyl oxygen atom on PET establishes a hydrogen bond with the hydroxyl hydrogen atom on the alcohol at around 0.2 nm, the distribution of distinct alcohol molecules at 0.2 nm near the PET chain was analyzed at molar ratio ranges of CO_2_ to ethanol of 0 and 0.5, as shown in Figure 10c,d. The findings revealed that the shorter the alcohol alkyl chain, the greater the distribution number near the PET chain. Simultaneously, upon the introduction of CO_2_ into the solution, the distribution values of alcohol molecules exhibited a corresponding drop. In comparison to long-chain alcohols, short-chain alcohols dissolve into the PET bulk phase more readily. Conversely, the introduction of CO_2_ replaced the alcohol molecules in their original position, leading to a reduction in the quantity of alcohol molecules present.

**Figure 11:**
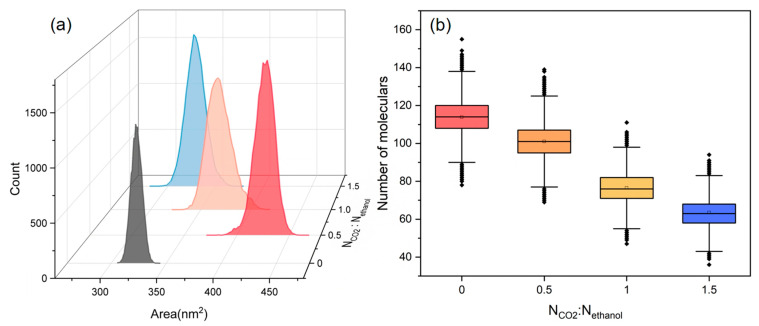
(**a**) The SASA distribution of PET, and (**b**) the number distribution of ethanol molecules within 0.2 nm proximity to PET at various CO_2_ contents.

The SASA of PET and the distribution value of ethanol molecules at 0.2 nm around PET were analyzed for the ethanol–PET–CO_2_ system at various CO_2_ contents, as shown in Figure 11a,b. The swelling impact of ethanol on PET tends to increase and subsequently diminish as the CO_2_ content increases, whereas the number of ethanol molecules scattered around PET continues to decrease. When considering the equilibrium snapshots (Figure 6), it is evident that the system exhibited clear phase separation and a significant influx of ethanol molecules into the CO_2_ phase in the presence of abundant CO_2_. Simultaneously, the PET chains experienced a reduction in volume due to the diminished ability of CO_2_ and ethanol molecules to dissolve PET. This is comparable to the trend shown in Figure 1, where the conversion of PET increases and subsequently decreases when ethanol is used. Thus, on a large scale, the addition of excessive CO_2_ does not consistently improve the alcoholysis process of PET.

## 4. Conclusions

In this study, the alcoholic behaviors of PET depolymerization when using various monohydric alcohols combined with ScCO_2_ have been investigated by using MD and experimental methods. The experimental results indicate that, in comparison to other alcohols, ScCO_2_ can be utilized to accelerate the ethanolysis of PET in a milder environment. PET can be completely depolymerized at a temperature of 473 K, a reaction time of 60 min, a molar ratio of CO_2_ to ethanol of 0.2, and a reaction pressure of 6 MPa. Under the combined effect of CO_2_ and ethanol, the random scission of PET dominated the early stage of the reaction, whereas the specific scission of PET dominated the later stage.

Furthermore, the MD simulation results show that ScCO_2_ improves the mass transfer process of depolymerization by accelerating the diffusion behavior of molecules in the system and increasing the swelling degree of the PET chain. The RDF results revealed that the hydrogen atoms on the hydroxyl group of the alcohols in the ternary system preferentially solvated the oxygen atoms on the PET’s carbonyl group, resulting in a greater hydrogen bonding effect. However, the excessive introduction of CO_2_ resulted in a reduction in the swelling degree of PET chains and a weakening of the hydrogen bonding between the alcohol molecules and the chains. This ultimately led to a decrease in the efficiency of the depolymerization process. The conclusive simulation results align with the experimental findings, confirming the enhancement mechanism of supercritical carbon dioxide during the PET alcoholysis process. Consequently, a novel strategy for the effective recycling of waste PET is proposed in this work.

## Figures and Tables

**Table 1:** Simulation cases with different compositions of binary and ternary systems.

Entry	Number of PET Chain	Composition	Molar Ratio (CO_2_/Alcohol)
1	6	methanol	0
2	6	CO_2_ + methanol	0.5
3	6	ethanol	0
4	6	CO_2_ + ethanol	0.5
5	6	CO_2_ + ethanol	1
6	6	CO_2_ + ethanol	1.5
7	6	1-propanol	0
8	6	CO_2_ + 1propanol	0.5
9	6	1-butanol	0
10	6	CO_2_ + 1-butanol	0.5
11	6	1-pentanol	0
12	6	CO_2_ + 1pentanol	0.5

**Table 2:** Average molecular weight (Mn) of residual PET at different reaction times, N_CO2_:N_ethanol_ = 0 and 0.2.

Reaction Time (min)	Mn [g/mol] (N_CO2_:N_ethanol_ = 0)	Mn [g/mol] (N_CO2_:N_ethanol_ = 0.2)
0	12,586	12,586
20	10,083	10,227
30	9791	1143
40	5796	750
50	2771	580
60	1889	546

**Table 3:** Hydrogen bonding interaction in PET–CO_2_–ethanol system at different CO_2_ contents.

	HBs Donor-Accepter	N_CO2_:N_ethanol_			
		0	0.5	1.0	1.5
Average Number of HBs	ethanol-PET	197.825	178.834	135.765	113.363
	ethanol-CO_2_	0	14.504	26.180	26.207
Average Lifetime of HBs [ps]	ethanol-PET	1.54	1.49	1.5	1.5
	ethanol-CO_2_	0	0.54	0.53	0.52

**Table 4:** Hydrogen bonding interaction between PET and alcohol molecules.

	N_CO2_:N_ethanol_	Type of Alcohol Molecules				
		Methanol	Ethanol	1-Propanol	1-Butanol	1-Pentanol
Average Number of HBs	0	201.76	197.825	190.883	186.988	186.295
	0.5	184.735	178.834	171.083	164.405	159.688
Average Lifetime of HBs [ps]	0	1.41	1.54	1.57	1.59	1.61
	0.5	1.4	1.49	1.52	1.54	1.57

## Data Availability

The data presented in this study are available upon request from the corresponding author.

## References

[1] Sun Y., Liu S., Wang P., Jian X., Liao X., Chen W.-Q. (2022). China’s Roadmap to Plastic Waste Management and Associated Economic Costs. J. Environ. Manag..

[2] Nisticò R. (2020). Polyethylene Terephthalate (PET) in the Packaging Industry. Polym. Test..

[3] IHS Markit (2018). Plasticizers. Chemical Economics Handbook.

[4] Korley L.T.J., Epps T.H., Helms B.A., Ryan A.J. (2021). Toward Polymer Upcycling—Adding Value and Tackling Circularity. Science.

[5] Nandi S., Mahish S.S., Das S.K., Datta M., Nath D. (2023). A Review of Various Recycling Methods of PET Waste: An Avenue to Circularity. Polym.-Plast. Technol. Mater..

[6] Chen R., Deng S., Cui T., Duan S., Jia Q., Zhang L. (2024). Progress in Recycling and Reutilization of Waste Polyethylene Terephthalate. Prog. Rubber Plast. Recycl. Technol..

[7] Martín A.J., Mondelli C., Jaydev S.D., Pérez-Ramírez J. (2021). Catalytic Processing of Plastic Waste on the Rise. Chem.

[8] Jehanno C., Alty J.W., Roosen M., De Meester S., Dove A.P., Chen E.Y.-X., Leibfarth F.A., Sardon H. (2022). Critical Advances and Future Opportunities in Upcycling Commodity Polymers. Nature.

[9] Barnard E., Rubio Arias J.J., Thielemans W. (2021). Chemolytic Depolymerisation of PET: A Review. Green Chem..

[10] Goto M. (2009). Chemical Recycling of Plastics Using Sub- and Supercritical Fluids. J. Supercrit. Fluids.

[11] Goto M., Jin F., Zhou Q., Wu B. (2010). Supercritical Water Process for the Chemical Recycling of Waste Plastics. AIP Conf. Proc..

[12] Sako T., Sugeta T., Otake K., Nakazawa N., Sato M., Namiki K., Tsugumi M. (1997). Depolymerization of Polyethylene Terephthalate to Monomers with Supercritical Methanol. J. Chem. Eng. Jpn. JCEJ.

[13] De Castro R.E.N., Vidotti G.J., Rubira A.F., Muniz E.C. (2006). Depolymerization of Poly(Ethylene Terephthalate) Wastes Using Ethanol and Ethanol/Water in Supercritical Conditions. J. Appl. Polym. Sci..

[14] Lozano-Martinez P., Torres-Zapata T., Martin-Sanchez N. (2021). Directing Depolymerization of PET with Subcritical and Supercritical Ethanol to Different Monomers through Changes in Operation Conditions. ACS Sustain. Chem. Eng..

[15] Nunes C.S., da Silva M.J.V., da Silva D.C., dos R. Freitas A., Rosa F.A., Rubira A.F., Muniz E.C. (2014). PET Depolymerisation in Supercritical Ethanol Catalysed by [Bmim][BF_4_]. RSC Adv..

[16] Imran M., Kim B.-K., Han M., Cho B.G., Kim D.H. (2010). Sub- and Supercritical Glycolysis of Polyethylene Terephthalate (PET) into the Monomer Bis(2-Hydroxyethyl) Terephthalate (BHET). Polym. Degrad. Stab..

[17] Barboza E.S., Lopez D.R., Amico S.C., Ferreira C.A. (2009). Determination of a Recyclability Index for the PET Glycolysis. Resour. Conserv. Recycl..

[18] Ding J., Chen J., Ji Y., Ni P., Li Z., Xing L. (2014). Kinetics of Alcoholysis of Poly(Ethylene Terephthalate) in Sub- and Super-Critical Isooctyl Alcohol to Produce Dioctyl Terephthalate. J. Anal. Appl. Pyrolysis.

[19] Liu F., Chen J., Li Z., Ni P., Ji Y., Meng Q. (2013). Alcoholysis of Poly(Ethylene Terephthalate) to Produce Dioctyl Terephthalate with Sub- and Super-Critical Isooctyl Alcohol. J. Anal. Appl. Pyrolysis.

[20] Liu J., Qin Z., Wang G., Hou X., Wang J. (2003). Critical Properties of Binary and Ternary Mixtures of Hexane+ Methanol, Hexane+ Carbon Dioxide, Methanol+ Carbon Dioxide, and Hexane+ Carbon Dioxide+ Methanol. J. Chem. Eng. Data.

[21] Pöhler H., Kiran E. (1997). Volumetric Properties of Carbon Dioxide+ Ethanol at High Pressures. J. Chem. Eng. Data.

[22] Liu J., Yin J. (2022). Carbon Dioxide Synergistic Enhancement of Supercritical Methanol on PET Depolymerization for Chemical Recovery. Ind. Eng. Chem. Res..

[23] Yu K., Liu J., Sun J., Shen Z., Yin J. (2023). Study of Polyester Degradation by Sub/Supercritical Ethanol and Enhancement of Carbon Dioxide. J. Supercrit. Fluids.

[24] Li X.-K., Lu H., Guo W.-Z., Cao G.-P., Liu H.-L., Shi Y.-H. (2015). Reaction Kinetics and Mechanism of Catalyzed Hydrolysis of Waste PET Using Solid Acid Catalyst in Supercritical CO_2_. AIChE J..

[25] Guo W.-Z., Lu H., Li X.-K., Cao G.-P. (2016). Tungsten-Promoted Titania as Solid Acid for Catalytic Hydrolysis of Waste Bottle PET in Supercritical CO2. RSC Adv..

[26] Spyriouni T., Boulougouris G.C., Theodorou D.N. (2009). Prediction of Sorption of CO_2_ in Glassy Atactic Polystyrene at Elevated Pressures through a New Computational Scheme. Macromolecules.

[27] Bozorgmehr M.R., Morsali A., Beyramabadi S.A., Moghaddam F.K., Pashirepour J., Shakeri M. (2014). All Atom Molecular Dynamics Simulation Study of Polyethylene Polymer in Supercritical Water, Supercritical Ethanol and Supercritical Methanol. J. Supercrit. Fluids.

[28] Sun F., Dedong H., Fei L., Weiqiang W., Zhaotao G., Zhuo Z. (2022). Molecular-Level Investigation of Plasticization of Polyethylene Terephthalate (PET) in Supercritical Carbon Dioxide via Molecular Dynamics Simulation. R. Soc. Open Sci..

[29] Yan M., Yang Y., Shen T., Grisdanurak N., Pariatamby A., Khalid M., Hantoko D., Wibowo H. (2023). Effect of Operating Parameters on Monomer Production from Depolymerization of Waste Polyethylene Terephthalate in Supercritical Ethanol. Process Saf. Environ. Prot..

[30] Abraham M.J., Murtola T., Schulz R., Páll S., Smith J.C., Hess B., Lindahl E. (2015). GROMACS: High Performance Molecular Simulations through Multi-Level Parallelism from Laptops to Supercomputers. SoftwareX.

[31] Potoff J.J., Siepmann J.I. (2001). Vapor–Liquid Equilibria of Mixtures Containing Alkanes, Carbon Dioxide, and Nitrogen. AIChE J..

[32] Chen B., Potoff J.J., Siepmann J.I. (2001). Monte Carlo Calculations for Alcohols and Their Mixtures with Alkanes. Transferable Potentials for Phase Equilibria. 5. United-Atom Description of Primary, Secondary, and Tertiary Alcohols. J. Phys. Chem. B.

[33] Wang J., Wolf R.M., Caldwell J.W., Kollman P.A., Case D.A. (2004). Development and Testing of a General Amber Force Field. J. Comput. Chem..

[34] Tian Lu Sobtop Version 3.1. http://sobereva.com/soft/Sobtop/.

[35] Lu T., Chen F. (2012). Multiwfn: A Multifunctional Wavefunction Analyzer. J. Comput. Chem..

[36] Berthelot D. (1898). Sur Le Mélange Des Gaz. C. R..

[37] Lorentz H.A. (1881). Ueber Die Anwendung Des Satzes Vom Virial in Der Kinetischen Theorie Der Gase. Ann. Der Phys..

[38] Bussi G., Donadio D., Parrinello M. (2007). Canonical Sampling through Velocity Rescaling. J. Chem. Phys..

[39] Berendsen H.J.C., Postma J.P.M., van Gunsteren W.F., DiNola A., Haak J.R. (1984). Molecular Dynamics with Coupling to an External Bath. J. Chem. Phys..

[40] Parrinello M., Rahman A. (1981). Polymorphic Transitions in Single Crystals: A New Molecular Dynamics Method. J. Appl. Phys..

[41] Essmann U., Perera L., Berkowitz M.L., Darden T., Lee H., Pedersen L.G. (1995). A Smooth Particle Mesh Ewald Method. J. Chem. Phys..

[42] Hess B., Bekker H., Berendsen H.J.C., Fraaije J.G.E.M. (1997). LINCS: A Linear Constraint Solver for Molecular Simulations. J. Comput. Chem..

[43] Humphrey W., Dalke A., Schulten K. (1996). VMD: Visual Molecular Dynamics. J. Mol. Graph..

[44] Martínez L., Andrade R., Birgin E.G., Martínez J.M. (2009). PACKMOL: A Package for Building Initial Configurations for Molecular Dynamics Simulations. J. Comput. Chem..

[45] Kumar R., Schmidt J.R., Skinner J.L. (2007). Hydrogen Bonding Definitions and Dynamics in Liquid Water. J. Chem. Phys..

[46] Asakuma Y., Yamamura Y., Nakagawa K., Maeda K., Fukui K. (2011). Mechanism of Depolymerization Reaction of Polyethylene Terephthalate: Experimental and Theoretical Studies. J. Polym. Env..

[47] Mehl A., Nascimento F.P., Falcão P.W., Pessoa F.L.P., Cardozo-Filho L. (2011). Vapor-Liquid Equilibrium of Carbon Dioxide + Ethanol: Experimental Measurements with Acoustic Method and Thermodynamic Modeling. J. Thermodyn..

[48] Chiu H.-Y., Lee M.-J., Lin H. (2008). Vapor-liquid Phase Boundaries of Binary Mixtures of Carbon Dioxide with Ethanol and Acetone. J. Chem. Eng. Data.

[49] Joung S.N., Yoo C.W., Shin H.Y., Kim S.Y., Yoo K.-P., Lee C.S., Huh W.S. (2001). Measurements and Correlation of High-Pressure VLE of Binary CO_2_–Alcohol Systems (Methanol, Ethanol, 2-Methoxyethanol and 2-Ethoxyethanol). Fluid Phase Equilibria.

[50] Liu C., Ling Y., Wang Z., Zheng W., Sun W., Zhao L. (2022). Unveiling the Microenvironments between Ionic Liquids and Methanol for Alcoholysis of Poly(Ethylene Terephthalate). Chem. Eng. Sci..

[51] Kazarian S.G., Vincent M.F., Bright F.V., Liotta C.L., Eckert C.A. (1996). Specific Intermolecular Interaction of Carbon Dioxide with Polymers. J. Am. Chem. Soc..

[52] Drohmann C., Beckman E.J. (2002). Phase Behavior of Polymers Containing Ether Groups in Carbon Dioxide. J. Supercrit. Fluids.

[53] Pardal F., Tersac G. (2006). Comparative Reactivity of Glycols in PET Glycolysis. Polym. Degrad. Stab..

[54] Aida T., Aizawa T., Kanakubo M., Nanjo H. (2010). Relation between Volume Expansion and Hydrogen Bond Networks for CO_2_-alcohol Mixtures at 40 °C. J. Phys. Chem. B.

[55] Suzuki T., Tsuge N., Nagahama K. (1991). Solubilities of Ethanol, 1-Propanol, 2-Propanol and 1- Butanol in Supercritical Carbon Dioxide at 313 K and 333 K. Fluid Phase Equilibria.

[56] Zheng W., Liu C., Wei X., Sun W., Zhao L. (2023). Molecular-Level Swelling Behaviors of Poly (Ethylene Terephthalate) Glycolysis Using Ionic Liquids as Catalyst. Chem. Eng. Sci..

[57] Miller-Chou B.A., Koenig J.L. (2003). A Review of Polymer Dissolution. Prog. Polym. Sci..

[58] Gurina D.L., Budkov Y.A., Kiselev M.G. (2022). Polylactide Nanoparticle Impregnation with Carbamazepine in Supercritical Media and Its Subsequent Release in Liquid Solvents: Insights from Molecular Simulation. J. Mol. Liq..

[59] Hirogaki K., Tabata I., Hisada K., Hori T. (2005). An Investigation of the Interaction of Supercritical Carbon Dioxide with Poly(Ethylene Terephthalate) and the Effects of Some Additive Modifiers on the Interaction. J. Supercrit. Fluids.

